# Nevi and Melanoma in Children: What to Do in Daily Medical Practice: Encyclopedia for Pediatricians and Family Doctors

**DOI:** 10.3390/diagnostics14182004

**Published:** 2024-09-10

**Authors:** Patrycja Sosnowska-Sienkiewicz, Danuta Januszkiewicz-Lewandowska, Jacek Calik, Gabriela Telman-Kołodziejczyk, Przemysław Mańkowski

**Affiliations:** 1 Department of Pediatric Surgery, Traumatology and Urology, Poznan University of Medical Sciences, 60-572 Poznan, Poland; mankowskip@ump.edu.pl; 2 Department of Pediatric Oncology, Hematology and Transplantology, Poznan University of Medical Sciences, 60-572 Poznan, Poland; danuta.januszkiewicz@ump.edu.pl (D.J.-L.); gabriela.telman@gmail.com (G.T.-K.); 3 Department of Clinical Oncology, Wroclaw Medical University, 50-556 Wrocław, Poland; jacek.calik@oldtownclinic.pl

**Keywords:** children, diagnostics, malignant melanoma, melanocytic nevus, treatment

## Abstract

Melanocytic nevi, commonly known as moles, are benign skin lesions that often occur in children and adolescents. Overall, they are less common in children compared to adults. Understanding the diagnosis and management of melanocytic nevi and risk factors for melanoma development is crucial for their early detection and appropriate treatment. This paper presents children’s most common melanocytic nevi, including their epidemiology, morphology, diagnostic methods, and treatment.

## 1. Introduction

A melanocytic nevus, or simply a nevus, is a benign skin lesion resulting from melanocyte proliferation. Nevi can take various forms, such as moles, spots, or nodules, and vary in color, including brown, black, red, pink, or blue. The appearance of nevi can range from single to multiple lesions and can also vary in size [1]. The number of nevi is the most potent risk factor for melanoma, especially when they appear at a younger age. Overall, nevi are less common in children compared to adults. They tend to develop and increase in number throughout childhood and adolescence, reaching peak prevalence in adulthood [1,2].

In the study of skin lesions, it is critical to differentiate between benign nevi and melanoma due to their very different prognoses. Melanocytes, pigment-producing cells in the skin, are derived from the neural crest during embryonic development. Neural crest markers are used in research and diagnostics to identify these cells. These markers are expressed in benign nevi and melanoma, but their patterns and levels of expression can differ, providing valuable information for diagnosis and understanding melanoma progression.

Both genetic and environmental factors play a role in developing nevi and melanoma [1,2].

Only 20–30% of melanomas are associated with nevi, but the differences between nevus-associated and de novo melanomas remain unclear. UV-induced mutations in BRAF or NRAS genes activate the MAPK pathway, and melanocytes undergo proliferation. Genome-wide association studies (GWASs) have identified various loci strongly associated with nevus number. Among them are IRF4 genes associated with pigmentation and melanocyte proliferation, MTAP involved in polyamine metabolism, DOCK8 regulating signal transduction, KIT-ligand (KITLG) acting in melanocyte development and melanin synthesis, and PLA2G6 playing a key role in regulating cell membrane homeostasis [3]. Studies have revealed a multi-gene predisposition pattern in which both rare high-penetration variants and common low-penetration variants modulate an individual’s risk of developing melanoma. Rare mutations in high penetrance genes are responsible for melanoma symptoms in about 22% of melanoma families (19% for CDKN2A and 3% for other genes), while the significant melanoma genetic factors underlying melanoma in melanoma-prone families are still unknown [3]. Next-generation sequencing has made it possible to identify high-risk melanoma variants in genes related to cell cycle control, such as BAP1, and in genes related to the telomere maintenance pathway, such as TERT, and the protective complex genes POT1, TERF2IP, and ACD. CDKN2A germline mutations remain the most critical risk factor for melanoma development and increase the risk of melanoma growth by up to 75 times under the influence of environmental risk factors such as UV exposure and sunburn [4,5]. Population-based GWASs have identified allelic variants in moderate- and low-risk genes for melanoma predisposition. They are involved in multiple biological pathways related to genesis (MTAP, PLAG2G6, and ATM genes), telomere maintenance (TERT, OBFC1, PARP1, and FTO genes), and pigmentation (MITF, OCA2, MC1R, and SLC45A2 genes), highlighting the complexity of melanoma skin etiology [4,5].

This paper presents the most common congenital and acquired benign melanocytic nevi in children, including their epidemiology, morphology, diagnostic methods, and treatment in the daily medical practice of pediatricians, family doctors, surgeons, and dermatologists. Special attention was paid to the possibility of melanomagenesis.

## 2. Diagnosis of Nevi

Before discussing the diagnosis of nevi in detail, it is worth emphasizing the difference between melanoma simulators and precursors, which is extremely important for the urgency of appropriate management.

Melanoma simulators are conditions or lesions that may resemble melanoma but are not themselves precursors to or directly associated with melanoma development. They can be benign lesions, such as moles (birthmarks), that share some characteristics with melanoma but do not have the potential to develop into melanoma. Simulators can sometimes present a diagnostic challenge because they may have features overlapping with melanoma, requiring careful evaluation by a dermatologist or healthcare professional [6,7,8].

Melanoma precursors are lesions or conditions that are directly related to the development of melanoma. Examples of precursors include dysplastic nevi. These lesions may have some atypical features on microscopic examination or may show signs of developing into invasive melanoma over time [6,7,8]. In summary, simulators are benign conditions that may resemble melanoma but are not associated with melanoma development, while precursors are.

**Figure 1:**
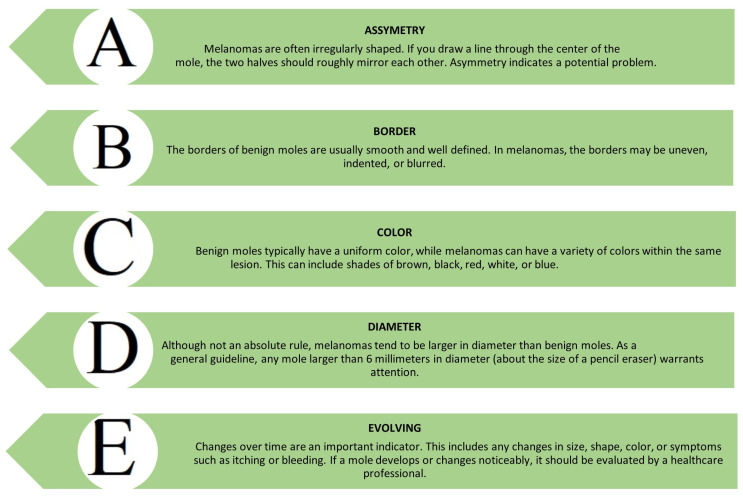
The ABCDE rule for melanoma skin cancer diagnosis.

The diagnosis of nevi is usually performed by a dermatologist or pediatric surgeon. The first and most crucial stage is a physical examination and a detailed visual assessment using the ABCDE rule. During the examination, the doctor evaluates the characteristics of the nevus, including its location, number, size, shape, color, and any changes over time [6]. The ABCDE rule is used to evaluate the possible malignant transformation of melanocytic nevi. The ABCDE rule includes A (asymmetry)—asymmetry; B (borders)—the edge is uneven and not sharp; C (color)—color—diverse with uneven distribution of the dye; D (diameter)—a diameter > 5 mm, with an increase in the diameter of the nevus; and E (elevation)—emphasizing the surface (Figure 1) [7,8].

The patient’s medical history should be considered during the examination. The healthcare provider will ask about risk factors such as a family history of melanoma, a personal history of sunburns, and the presence of dysplastic moles. Any symptoms associated with the mole or skin lesion, such as itching, bleeding, or changes over time, will be discussed.

Current methods for evaluating nevi should include dermoscopy and 2D/3D digital imaging. Dermoscopy, also known as dermatoscopy or epiluminescence microscopy, is a non-invasive technique that allows the skin to be examined under magnification. Dermoscopy helps visualize structures and patterns within a nevus and makes it easier to distinguish a malignant lesion from a benign one [9]. Serial 2D or 3D digital imaging is becoming more widely used. It is designed to monitor changes in the nevus over time.

If there are concerns about changes affecting, for example, the nevus, an excisional biopsy with a histopathologic examination and evaluation of molecular changes is indicated [6,9]. The biopsy sample involves analyzing the cells and tissues to determine if they are cancerous and, if so, the type and stage of melanoma [6,9].

## 3. Congenital Nevus

A congenital nevus appears at birth or in the first year of life and is usually single, oval, and uniform in color. It is a neural crest-derived hamartoma determined in utero. This type of nevus is a rare melanocytic lesion with a large size, papillary or nodular surface, and regular color, often associated with the presence of hair [2].

The classification of congenital nevi can be based on their size and characteristics, and they are typically categorized as small, medium, or large/giant congenital nevi. The American Academy of Dermatology generally uses the following criteria for classification:

- Small congenital nevi: these are less than 1.5 cm in diameter.
- Medium congenital nevi: these range from 1.5 to 19.9 cm in diameter.
- Large/giant congenital nevi: 20 cm or larger in diameter [10].

Another consensus-based classification of congenital melanocytic nevi includes additional categories based on expected adult size, location (head, trunk, and extremities), the number of satellite nevi, and morphological features (color heterogeneity, roughness, nodularity, and hypertrichosis). This classification system allows for an easy and detailed phenotypic characterization of each congenital melanocytic nevus [5].

There have been several studies attempting to identify genetic hits underlying congenital nevi pathogenesis, and the aggregate results of these studies suggest that NRAS mutations and, to a lesser extent, BRAF mutations contribute to the development of congenital nevi [5,10]. Congenital nevi can be associated with nevus-associated melanoma and are also usually caused by an NRAS mutation. Large (more than 20 cm) nevi are associated with a 10–15% increased risk of melanoma [10].

The follow-up and monitoring of individuals with congenital nevi, regardless of their size, are essential due to the potential for these nevi to progress. The following should be checked in the follow-up:

- Regular skin examinations: Patients with congenital nevi should have regular skin examinations conducted by a dermatologist. The frequency of these examinations may vary based on the size and characteristics of the nevi but often starts in infancy and continues throughout life. For large/giant congenital nevi, more frequent monitoring is usually recommended.
- Changes in the nevi: Any changes in the size, color, shape, or texture should be reported to a healthcare professional promptly. This includes new symptoms such as itching, bleeding, or pain associated with the nevi.
- Photography: Dermatologists may use photography to document the appearance of congenital nevi over time. This can help in tracking changes that might occur and aid in the early detection of any concerning developments.
- Biopsy or excision: a dermatologist may sometimes recommend a biopsy or excision of a congenital nevus if there are any suspicious changes or if the nevus is particularly large or irregular in shape.
- Sun protection: Individuals with congenital nevi should practice suitable sun protection measures to reduce the risk of sun-induced damage to the skin. This includes using sunscreen with a high SPF, wearing protective clothing, and avoiding excessive sun exposure.
- Counseling and education: patients and their families should receive counseling and education about the condition, the importance of monitoring, and the signs of melanoma.

**Figure 2:**
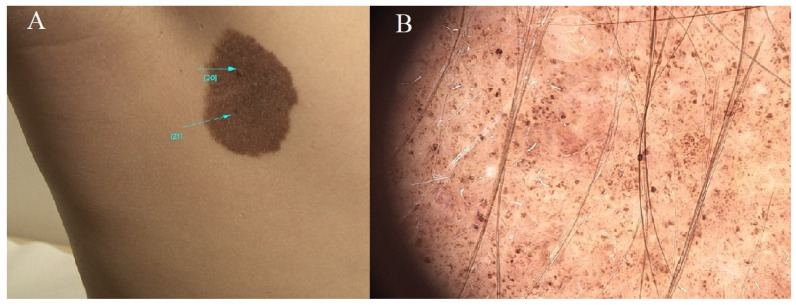
Congenital nevus. (**A**) Ellipsoid-shaped brown nevus measuring approximately 5 cm in length. Uniform brown pigmentation. Within the lesion, isolated papillated nodules in concordance with the nevus’ hue are evident. (**B**) Nevus demonstrating a nodular pattern with a harmonious structure and coloration. Terminal hairs are discernible within the confines of the lesion. Blue lines indicate the lesion being examined, magnification 60×.

It is essential to remember that most congenital nevi do not become cancerous, and many people with these nevi lead healthy lives. However, regular monitoring and early detection are crucial in managing the potential risks associated with these nevi. A consultation with a dermatologist is critical to developing an appropriate follow-up plan tailored to an individual’s circumstances [10] (Figure 2).

## 4. Acquired Melanocytic Nevi

Acquired melanocytic nevi usually develop within the first 20 years of life and are most common in people of European ethnicity. They occur as benign and dysplastic lesions. Acquired nevi share the same genetic risk factors as malignant melanoma. They have a higher rate of BRAF hotspot mutations and a lower rate of NRAS hotspot mutations compared to congenital nevi.

### 4.1. Benign Melanocytic Nevus

Benign nevi appear mainly during adolescence. They are characterized by a regular, symmetrical structure, a well-defined border, and evenly distributed pigment. The risk of developing melanoma from such acquired benign nevi is very low [1,10]. This lesion requires periodic dermatological observation (follow-up every 12 months) and sun protection. In case of any doubt, a diagnostic excisional biopsy is recommended.

**Figure 3:**
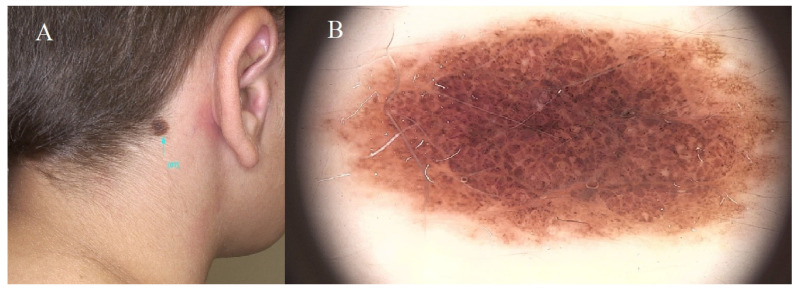
Acquired melanocytic nevi. (**A**) Marginally raised brown nevus situated within the hair-bearing area of the neck. (**B**) Nevus displaying a nodular pattern with congruous structural and chromatic attributes. The central region exhibits dark brown nodules, while the periphery features lighter brown nodules. The blue line indicates the lesion being examined, magnification 20×.

It is important to remember that while most common moles are benign, changes in size, shape, color, or other unusual features should be evaluated by a dermatologist to rule out the possibility of melanoma or other skin conditions. Additionally, individuals should regularly monitor their moles and protect their skin from excessive sun exposure to reduce the risk of skin cancer [1,10,11] (Figure 3).

### 4.2. Dysplastic Nevus (Atypical Nevus)

Dysplastic nevus, clinically called atypical nevus, is characterized by its asymmetrical shape, size of more than 5 mm, irregular edge, and variety of colors. Dysplastic nevi can have a mixture of colors, such as brown, pink, and tan shades. This color diversity can give them a mottled or speckled appearance. They can be flat, slightly raised, or have a bumpier surface. Some may appear elevated compared to the surrounding skin. This type of nevus can develop into melanoma. Some individuals have a family history of dysplastic nevi, and these individuals may be at a slightly higher risk of melanoma. However, most dysplastic nevi occur sporadically. The presence of numerous dysplastic nevi is sometimes called “dysplastic nevus syndrome”. Dysplastic nevus syndrome refers to patients with 50 or more dysplastic nevi. These patients develop dysplastic nevi during adolescence and adulthood. A patient is said to have dysplastic nevus syndrome if they have five or more dysplastic melanocytic nevi. Dysplastic nevi show an upregulation of follicular keratinocyte-related genes compared to common nevi. Anatomical locations and DNA signatures of nevi implicate ultraviolet radiation and non-ultraviolet radiation pathways in nevogenesis.

There is a variant of dysplastic nevus, described in the early 1990s, known as lentiginous dysplastic nevus of the elderly. Some consider this to be nevoid lentigo maligna or evolving or early melanoma in situ in older people. These nevi appear in elderly individuals older than 60 years and tend to appear on the back in men and on the legs in women.

Rarely, dysplastic nevi may present in an eruptive form, primarily in immunosuppressed patients [2,12].

**Figure 4:**
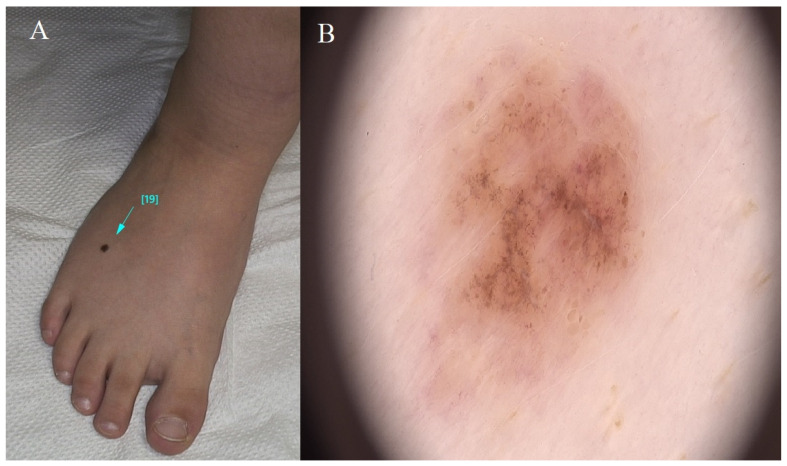
Dysplastic nevus. (**A**) Flat brown lesion located on the dorsal surface of the foot. (**B**) Pigmented lesion featuring a nodular pattern and homogenous brown pigmentation. Nodules of variable dimensions and shades are dispersed haphazardly. Peripheral amorphous pinkish zones denote increased vascularity. The blue line indicates the lesion being examined, magnification 20×.

It is important to remember that the presence of dysplastic nevi does not guarantee the development of melanoma, and most dysplastic nevi remain benign. However, given their increased risk of melanoma and the potential for change, individuals with dysplastic nevi should be vigilant about skin self-examinations and follow-up with a dermatologist as recommended. Therefore, constant frequent dermatological observations (control every 6 months) and sun protection are required. In case of any doubt, a diagnostic excisional biopsy is indicated [12] (Figure 4).

## 5. Other Nevi

Nevus spilus (speckled lentiginous nevus) is clinically characterized by a light brown macular background dotted with many darker spots or papules. These speckles can be evenly distributed across the surface of the nevus. The term “nevus spilus” means “mole with spots” in Latin, and this name reflects the defining characteristic of the condition. This type of lesion can be acquired or congenital. It can affect any body part, but the chest and upper extremities are the most common locations. Combining a diffuse nevus with other hypo- or depigmented lesions is rare [13]. The development of melanomas within diffuse nevi has been reported. It is important to note that while nevus spilus is typically benign, any changes in size, shape, or color, or the development of symptoms such as itching, bleeding, or pain within the lesion, should prompt a visit to a dermatologist for evaluation. If this type of lesion is diagnosed, the child should undergo periodic dermatologic follow-up, and a diagnostic excisional biopsy is indicated if there are rapidly growing nodular lesions within the nevus or signs of proliferation [13].

Nevus spilus can present in two main variants: macular and papular. In the macular variant, the background lesion appears flat, with a tan or brown coloration covering a relatively large skin area. Scattered throughout the macule, smaller, darker speckles give it a “speckled” appearance. The papular variant features the same background macule but has raised or elevated papules on its surface. These papules can vary in size and may be darker or lighter in color compared to the surrounding macule [13].

The etiology of NS is not entirely clear, but mutations in HRAS have been implicated. Sequencing results have also indicated a somatic mutation in the gene of NRAS.

**Figure 5:**
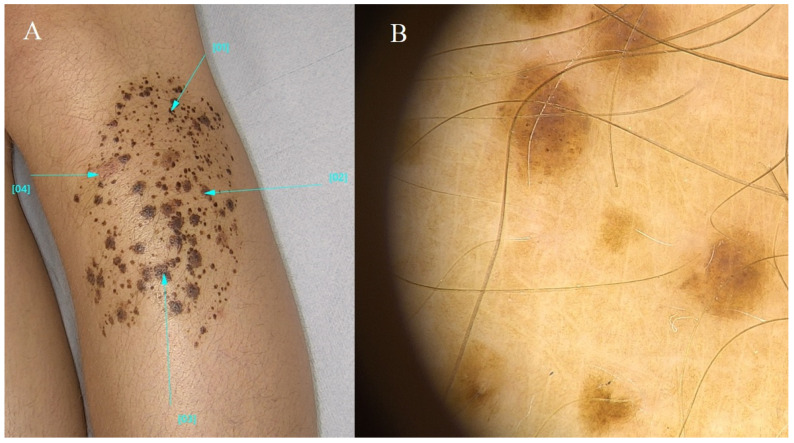
Nevus spilus. (**A**) Ellipsoidal pigmented lesion on the shin, approximately 10 cm in size, comprising heterogeneous colorations ranging from light brown to dark brown and gray. Pigmented regions are interspersed with unaffected skin. (**B**) Homogeneous nevus composed of numerous smaller areas exhibiting a reticular pattern and uniform brown hue. The described structures are interspersed amidst unaltered skin. Blue lines indicate the lesion being examined.

Nevus spilus usually occurs as an isolated lesion and is not associated with underlying syndromes or systemic conditions. Syndromic pigmentation refers to skin changes related to underlying genetic syndromes or systemic conditions. Examples of syndromes with associated pigmentation changes include neurofibromatosis type 1 (café-au-lait spots), tuberous sclerosis (hypopigmented macules or ash-leaf spots), and Peutz–Jeghers syndrome (multiple lentigines on the lips and oral mucosa). Syndromic pigmentation may present as isolated pigmented lesions or as part of a broader constellation of signs and symptoms characteristic of the underlying syndrome. Nevus spilus is a specific pigmented skin lesion characterized by a speckled appearance. At the same time, syndromic pigmentation refers to pigmented skin changes associated with underlying genetic syndromes or systemic conditions. Healthcare providers need to differentiate between these conditions to guide their appropriate management and surveillance [4,10,11,13] (Figure 5).

A spitz nevus (Spitz tumor) is also known as a spitzoid nevus or spindle and epithelioid cell nevus with histopathologic features of a Spitz nevus that ranges from benign to malignant. The current classification of a Spitz tumor includes benign Spitz nevi (SNs), an atypical Spitz nevus/tumor (AST) as a tumor of intermediate malignant potential, and Spitz melanoma (SM). The WHO 2018 guideline differentiates Spitz versus spitzoid as follows: Spitz lesions are associated with at least a single specific genetic alteration (*HRAS* mutation, fusions in *ROS1*, *ALK*, *NTRK1*, *NTRK3*, *MET*, and *RET*), and spitzoid lesions are lesions with a morphological resemblance to Spitz nevi. BRAF V600 mutations, the most common mutations in melanomas, are absent in Spitz tumors.

Clinically, Spitz melanomas are usually evolving amelanotic nodular lesions and can grow to a diameter of ≥1 cm; they can often go clinically undiagnosed because of their wide range of clinical appearances and their lack of pigmentation. Spitz melanoma is typically larger than an atypical Spitz tumor, with a mean diameter of 1 cm, and is more likely to be nodular than Spitz nevi but can also present as flat lesions. Spitz melanomas exhibit a wide range of colors, with 80% containing red/pink, 50% containing dark brown, 35% containing gray, and 33% containing light brown [14]. The accurate diagnosis of Spitz nevi usually requires dermatopathological expertise, and a consultation with a dermatologist is crucial for their appropriate management. The dermoscopic monitoring of this type of nevus should occur every 3–6 months when the lesion is less than 1 cm and has a typical dermoscopic presentation. Monitoring should continue in children under 12 until a homogeneous image is obtained upon dermoscopic examination. It should be removed when the Spitz nevus is more significant than 1 cm, nodular, ulcerated, and proliferating. In adults, Spitz nevi are an indication for excision [14].

Atypical Spitz tumors often metastasize to regional lymph nodes; however, they are rarely associated with further clinical progression. A sentinel lymph node biopsy does not seem relevant as a staging or prognostic procedure, and its indication remains debatable and controversial [15,16].

A spitz nevus is a benign melanocytic lesion that shares many histological features with melanoma. Good symmetry, Kamino bodies, and a side-to-side uniformity of cell sockets or sheets favor the Spitz mark. The presence of abnormal mitoses, a mitotic rate in the skin > 2/mm^2^, and mitotic numbers within 0.25 mm of the deep edge of the lesion favor melanoma. Immunohistochemical staining for HMB45 and Ki67 sometimes provides additional helpful information. Nevertheless, it may be impossible to make a precise diagnosis [15,16].

**Figure 6:**
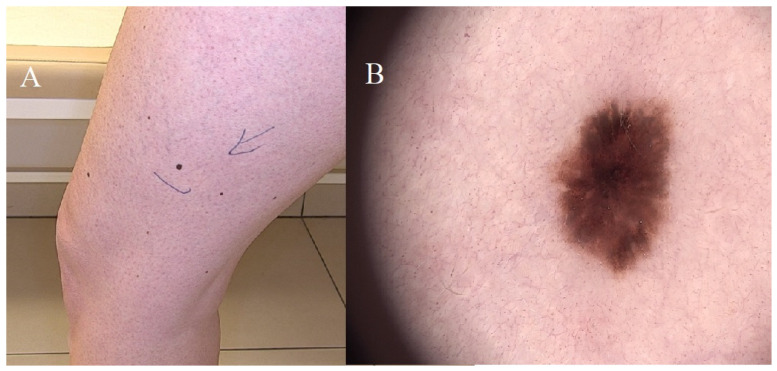
Spitz nevus. (**A**) Solitary flat brown lesion on the left thigh. (**B**) Lesion displaying a radial streak pattern. Disharmony in the lesion’s chromatic attributes is noted, with dark brown lines at the lesion’s periphery.

The exact rules of surgical and therapeutic procedures are thoroughly discussed in the article “ESP, EORTC, and EURACAN Expert Opinion: practical recommendations for the pathological diagnosis and clinical management of intermediate melanocytic tumors and rare related melanoma variants” [17] (Figure 6).

## 6. Reed Nevus

Reed described a pigmented spindle cell nevus as a variant of the Spitz nevus in 1975 [1]. He reported some differences between a pigmented spindle cell nevus and a classic Spitz nevus, including exclusive spindle cell composition, compressive rather than infiltrative growth, and abundant melanin with melanophages.

Reed nevus is characterized by a dark brown or black papule or plaque that most commonly affects the lower limbs of young females in the third decade of life; however, it can occur in children and young adults of both sexes. It is usually smaller than a Spitz nevus. It may proliferate. Less commonly, it may present as hypo- or amelanotic.

Characteristics of a Reed nevus in dermoscopy include the following: a dark brown to black color, a starburst pattern of pigmentation, radial lines or pseudopods that are distributed symmetrically around the entire circumference, dark and structureless in the center, occasionally, there are thick gray reticular lines centrally, and occasionally, black dots or clods are present peripherally.

Reed’s pigmented spindle cell nevus carries an NTRK translocation as a distinctive molecular feature.

Atypical lesions may be associated with a more aggressive clinical course, and it is difficult to distinguish these lesions from classic spitzoid melanoma. Therefore, it is recommended that late-onset lesions in adults with features of a Spitz nevus be surgically excised and sent for a histopathologic examination. The same is true for lesions with rapid and recent color, shape, and size changes. There is no consensus in the literature regarding the definition of surgical margins after excisional biopsy with a 1–2 mm margin and histopathologic confirmation. Some authors recommend extending the margin to 1 cm in the case of atypical lesions.

Children under 12 years of age with a classic starburst pattern upon dermoscopy may undergo clinical and dermoscopic follow-up. In these cases of typical, relatively small, symmetrical, non-ulcerated lesions located in classic areas and in places where surgical excision may cause cosmetic damage, careful follow-up may be performed every 3 to 6 months. In the absence of significant changes in the appearance of the lesion (color, shape, and size), this follow-up may be continued until a homogeneous pattern appears. After that, annual follow-up is recommended until the lesion has been wholly involutioned. It is suggested that all spitzoid lesions in children, palpable and erythematous, should be excised, especially those larger than 1 cm, nodular, ulcerated, and with a rapid evolution with changes in appearance. The excision of lesions in patients older than 12 years is also recommended, regardless of their atypical clinical or dermoscopic features [2,3,4].

Sutton’s nevus, also known as a halo nevus or leukoderma acquisitum centrifugum, is characterized by a depigmented or hypopigmented (lighter in color) ring or halo surrounding an existing mole (nevus). The central mole in a Sutton nevus is typically pigmented and may vary in color, such as brown or black. It is most common in children who have multiple moles. A return to normal coloration of the extensive hyperpigmentation is observed. There are no indications for the removal of typical Sutton nevi. Sutton nevi are generally benign and usually do not cause health problems. They are thought to result from an immune response targeting the nevus cells, leading to the loss of pigmentation in the surrounding skin. Dermatological follow-up is necessary only if one fears melanoma at the time of regression, which may mimic a Sutton nevus. An excisional biopsy should clarify any doubt [18,19].

A halo nevus does not require treatment unless there are concerning features or changes. Sometimes, the central pigmented lesion may regress spontaneously, leaving only the depigmented halo. The halo phenomenon may also occur around lesions with varying degrees of histologic atypia. Potential concern has been raised by reports of malignant melanoma exhibiting the halo phenomenon and the increased incidence of halo nevi in adults with melanoma. Cases of halo nevi associated with autoimmune hyperthyroidism and hypothyroidism have been reported, and halo nevi associated with nonsegmented vitiligo appear to be more common in patients with a family history of autoimmune disease. An increased incidence of halo nevi has also been observed in patients with Turner syndrome, suggesting a genetic basis for this tendency. The exact genetic mechanisms have not been identified.

It is important to note that the “halo sign” can sometimes be seen in association with melanoma, especially after an inflammation or regression of the lesion. This phenomenon may occur spontaneously after treatment of the melanoma. Therefore, careful clinical examination, imaging studies, and possibly biopsy may be necessary to assess the status of the lesion and guide further management [3,4,18].

**Figure 7:**
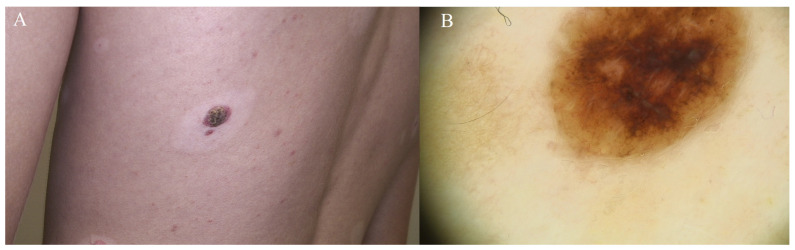
Sutton’s nevus. (**A**) Brown, raised nevus encircled by hypopigmented skin. (**B**) Concentric nevus showcasing a pattern characterized by both homogeneity and nodularity. Single gray nodules at the lesion’s periphery signify nevus regression, attributed to the presence of melanophages.

Halo nevus-like melanoma usually presents in late adulthood. The halo may be clinically (and histopathologically) asymmetric. In patients older than 45–50 years, excising any “halo” pigmented lesions is recommended (Figure 7).

**Figure 8:**
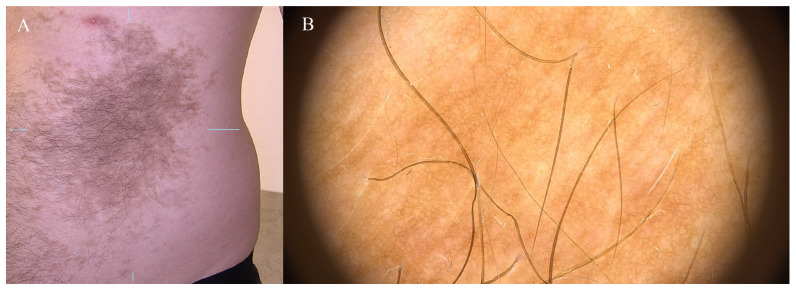
Becker’s nevus. (**A**) Extensive pigmented lesion on the abdominal integument, adorned with dark, coarser hairs than the surrounding epidermis. (**B**) Extensive nevus featuring a reticular line pattern and an abundance of hairs with hypopigmented follicular openings. Blue lines indicate the lesion being examined.

Becker’s nevus (Becker’s melanosis and Becker’s pigment hamartoma) is a large brown spot with increased hairiness (more common in young men). It develops in childhood or adolescence. This type of melanosis is a benign lesion that does not require special treatment and does not pose a risk of transformation to melanoma. The exact etiology is unclear. Associated features such as peri-pubertal development, male preponderance, hypertrichosis, and acneiform lesions suggest a role for androgens. Some investigators have described an increased number of androgen receptors in the lesional skin. Postzygotic mutations in beta-actin have been reported in association with both Becker’s nevus and Becker’s nevus syndrome. However, it should be appropriately diagnosed and subjected to periodic dermatological follow-up with dermoscopic examination [20]. Becker’s nevi are typically benign skin lesions, but they can be a source of cosmetic concern for some individuals due to their appearance or hair growth. Treatment options, such as laser therapy or hair removal, may be considered for cosmetic reasons in a consultation with a dermatologist. Unilateral hypomastia has been reported in association with a Becker’s nevus of the breast [2,20] (Figure 8).

The blue nevus, cellular blue nevus, or dermal melanocytoma is a maculopapular lesion the size of a lentil grain with a characteristic blue color, often present at birth or appearing in early childhood. The blue color can vary in intensity and may be more prominent in some cases than others. They are usually well circumscribed and have a round or oval shape. The borders are typically well defined. Blue nevi are often found on the skin, most commonly on the scalp, face, hands, and feet. They can also occur in other parts of the body. Additionally, blue nevi can occur on mucosal surfaces, such as the oral cavity or genital area. They belong to the so-called “safe nevi”, which are rarely the starting point of melanoma.

There are many types of this lesion, including the following: an ordinary blue nevus, cellular blue nevus, amelanotic blue nevus, mixed blue nevus, sclerosing (desmoplastic) blue nevus, epithelioid nevus, and subungual blue nevus.

The GNAQ and GNA11 proteins are considered the most critical molecules controlling early melanoblast proliferation in the dermis, and activating mutations in GNAQ and GNA11 cause a steady increase in melanoblast numbers. Somatic mutations in the GNAQ gene have been identified in about 80% of human blue nevi, 50% of MABNs (melanomas associated with a blue nevus), and more than 40% of uveal melanomas.

The resection of a blue nevus is unnecessary unless there are cosmetic concerns or signs of a malignant transformation. A surgical biopsy should be performed only in case of diagnostic doubt during dermoscopic examination [2,3]. It is important to remember that while blue nevi are generally benign, having any unusual or changing skin lesions evaluated by a dermatologist for an accurate diagnosis and appropriate management is crucial. Regular skin checks and sun protection measures are essential for overall skin health.

Although cellular blue nevi are usually benign, they can sometimes be confused with malignant skin lesions due to their pigmented appearance and cellular composition. However, they typically lack features of malignancy such as cytologic atypia, mitotic activity, or infiltrative growth patterns. Pigmented epithelioid melanocytoma (PEM) is a rare melanocytic tumor with intermediate malignant potential, meaning it has the potential for aggressive behavior but does not metastasize as readily as conventional melanoma. Clinically, it often presents as a pigmented papule, nodule, or plaque on the skin or mucosal surfaces. It can occur at any age but is more common in adults. Despite its intermediate malignant potential, pigmented epithelioid melanocytoma has a relatively indolent clinical course, with a low probability of systemic spread (2–3%) despite its ability to metastasize to regional lymph nodes (40–41%) or locally recur (7–8%) [11,21]. Because of the possibility of local recurrence, careful follow-up is recommended.

**Figure 9:**
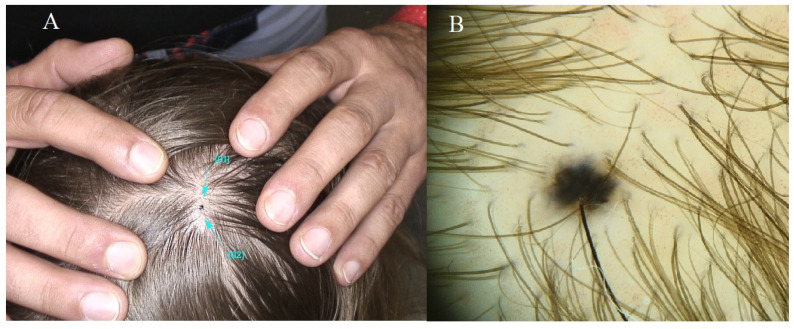
Blue nevus. (**A**) Small blue lesion, approximately 3 mm in diameter, localized within a pilosebaceous region of the skin. (**B**) Nevus displaying homogeneity in both color and structure, characterized by a blue hue. Terminal hairs are observed at the lower pole of the lesion, accompanied by hypopigmentation at the follicular orifices. Blue lines indicate the lesion being examined.

In conclusion, cellular blue nevus is a benign pigmented lesion characterized by spindle-shaped melanocytes and abundant blue-gray pigments. In contrast, pigmented epithelioid melanocytoma is a rare melanocytic tumor of intermediate malignant potential characterized by a proliferation of epithelioid melanocytes with cytologic atypia. Correctly diagnosing these lesions requires careful clinical and histopathologic evaluation to differentiate them from other pigmented lesions, including malignant melanoma [4,7,15]. The concept of morphologically intermediate melanocytic tumors or tumors with intermediate malignant potential is difficult to explain, and an attempt to clarify this issue was described by Ferrara et al. (2024) [22] (Figure 9).

The similarities and differences in each type of nevus are summarized in Table 1.

## 7. Melanoma in Children

Melanoma is the most malignant skin cancer that originates from melanocytes. It can rarely occur in children. Melanoma can arise de novo or along nevi. Nevus-associated melanoma is characterized by an occurrence at a younger age in people with a high number of nevi on the body, fair skin, red hair, location on the trunk, and moderate sun exposure. Multiple genetic alterations are associated with the development of melanoma. BRAF is the most commonly mutated gene in up to 50% of melanomas. NRAS is the second most frequently mutated oncogene in melanoma in up to 25% of cases. Mutations in the TERT gene promoter and silencing of the PTEN have been identified in primary and metastatic melanoma with a frequency of 30–85%. Concurrent BRAF and PTEN mutations have been rarely observed [4,23,24]. Melanoma can develop on desmoplastic, Spitz, blue, or congenital nevi. Other factors for the increased risk of melanoma development in children include a family history of melanoma, previous history of malignancy and bone marrow transplants, burns from excessive sun exposure, increased UV exposure, and sun-sensitive skin phenotype [4,25]. Children with Xeroderma pigmentosum (XP) and familial dysplastic multiple mole and melanoma (FAMMM) syndrome need special dermatology care [26]. Xeroderma pigmentosum is a rare autosomal recessive genodermatosis resulting from mutations in nucleotide excision repair. The condition characteristically demonstrates severe photosensitivity, skin pigmentary changes, malignant tumor development, and occasionally progressive neurologic degeneration.

Since the discovery of CDKN2A, many other genetic mutations responsible for melanoma predisposition have been identified. These include alterations in CDK4, ACD, BAP1, MITF, POT1, TERF2IP, and TERT. However, compared to CDKN2A mutations, which account for about 40 percent of familial atypical multiple mole–melanoma (FAMMM) syndrome and 2 percent of all melanoma cases, these subsequently discovered melanoma-predisposing mutations are relatively rare, comprising less than 1 percent of hereditary melanoma [4,26].

There are many classifications of childhood melanoma in the available literature. According to the WHO Classification of Skin Tumors (2018), cutaneous, mucosal, and uveal melanomas are distinguished based on the body part as the starting point.

**Figure 10:**
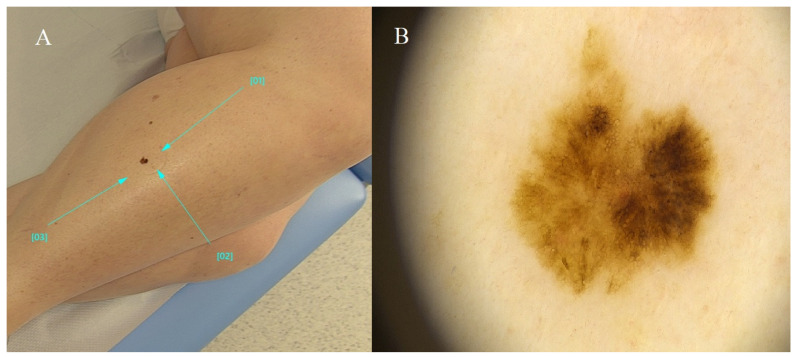
Superficial spreading melanoma. (**A**) Flat brown lesion on the skin of the right shin, measuring approximately 5 mm in diameter. (**B**) Nevus featuring an asymmetric reticular line pattern in both structural and chromatic aspects, with the presence of brown lines arranged segmentally and the observation of atypical reticular lines. Blue lines indicate the lesion being examined.

If nevi are common in children, melanomas remain the exception. They cannot be grouped into a single entity because they represent a variety of clinical situations, each with a different prognosis. For this reason, we often see the classification of melanoma in children as congenital melanoma, melanoma arising in congenital nevus, melanoma in systemic predisposing conditions, Spitz tumor/melanoma, and adult-type melanoma (conventional melanoma). Conventional melanoma is the most frequent type in children. Superficial spreading and nodular melanomas are the most frequent histological types among conventional melanoma cases, with a higher prevalence of nodular melanoma cases in children than in adults. Nodular melanomas constitute 40–50% of those in children [27,28]. Adult-type melanoma shares the exact causes and risk factors as in adults. It is also clinically and molecularly similar. Adult-type childhood melanoma is more often nodular than post-pubertal melanoma [27]. Nodular histological features are associated with reduced survival in children [28] (Figure 10).

Melanoma in children is similar to that in adults but has some distinct features. These include the rarity of occurrence and the specific location of lesions. Melanoma accounts for less than 1% of all cancers in children and occurs in unusual locations such as the palms of the hands, soles of the feet, and mucous membranes. The diagnosis of melanoma in children and adults is based on dermoscopic examination, with a histopathological analysis of the removed lesion. It is important to remember that a mutational analysis in melanoma can guide treatment but not diagnosis. The treatment of malignant melanoma in children usually involves a surgical removal of the tumor. When melanoma is suspected, the correct margin for an excisional biopsy is 1–3 mm. The results of fine- or core-needle aspiration biopsies and incisional or shear biopsies (shave biopsies) do not provide reliable information about the primary melanoma as needed. The American Joint Committee on Cancer/Union for International Cancer Control (AJCC/UICC) system should not be used. If a lesion is extensive and ulcerated, the material can be removed using imprint cytology (applying a glass slide to the tumor surface and material for cytologic examination) [3,4,10].

It is now known that certain subtypes of melanoma are associated with specific mutations (BRAF, KIT, and NRAS mutations). Genetic testing should be performed in quality-controlled centers [10]. If melanoma is confirmed, additional tests may be carried out to determine the extent of the disease (staging). This may include imaging tests such as CT scans or MRIs [4,10]. The status of nearby lymph nodes may also be evaluated because melanoma can spread to these nodes. Sentinel node biopsy is now an essential method for assessing the presence of micrometastases in the lymph nodes. Preoperative and intraoperative lymphoscintigraphy combined with staining should be used when performing sentinel node biopsy. Sentinel node biopsy should be performed after excisional biopsy of melanoma, at the same time as radical excision of the scar after excisional biopsy [1,3,4]. It is important to note that early detection is critical to successfully treating melanoma [1,3,4,7,8].

Additional treatments, such as immunotherapy or targeted therapy, are used for advanced forms of melanoma [22,28]. The National Comprehensive Cancer Network (NCCN) provides guidelines for treating melanoma, regularly updated based on the latest research and clinical evidence. A general overview of the treatment approaches for melanoma according to the NCCN guidelines includes surgery, adjuvant therapy, immunotherapy, targeted therapy, and radiation therapy. Surgery is the primary treatment for localized melanoma. The goal is to remove the tumor with a margin of healthy tissue around it to ensure its complete removal. The extent of surgery depends on the stage and location of the melanoma. Adjuvant therapy may be recommended for individuals with high-risk melanoma (Stage IIB, IIC, III, and some Stage IIA cases). Adjuvant therapy may include immunotherapy, targeted therapy, or participation in clinical trials. Immunotherapy helps the body’s immune system recognize and attack cancer cells. Checkpoint inhibitors such as pembrolizumab, nivolumab, and ipilimumab are commonly used immunotherapy drugs for melanoma. Targeted therapy is used for melanomas with specific genetic mutations, such as the BRAF mutation. Drugs like vemurafenib, dabrafenib, and trametinib target these mutations to inhibit cancer cell growth. Radiation therapy may be used as a primary treatment for melanoma in cases where surgery is not feasible or as an adjuvant treatment to surgery to reduce the risk of recurrence.

Participation in clinical trials may be recommended at any stage of melanoma treatment to access novel treatments or combinations of therapies being investigated. These general guidelines and treatment decisions should be personalized based on factors such as the stage of melanoma, the patient’s overall health, genetic factors, and preferences. Patients must discuss treatment options thoroughly with their healthcare team to make informed decisions [29].

Survival for melanoma in children has improved over the past 30 years. The five-year overall survival rate for all stages is 87–95%. Similar to adults, the main predictor of outcome in melanoma is the stage at diagnosis. Patients with Stage IV metastatic melanoma have a median survival of <1 year and a 5-year overall survival of <12%. Children <10 years of age are more likely to present with higher-stage disease, which may be due in part to the delayed diagnosis of children with melanoma. The prognostic role of age, gender, tumor thickness, ulceration, and sentinel lymph node status is difficult to assess. Similar to adults, a positive sentinel lymph node is associated with a worse prognosis. The prognostic implications of other traditional risk factors in adult melanoma, including vertical growth phase, vascular invasion, and high mitotic activity, do not appear to correlate with an increased risk of mortality [24].

## 8. Discussion

Nevi are common in children, just as they are in adults. They typically begin to appear in childhood and can continue to develop throughout a person’s life. Some moles may occur at birth (congenital nevi), while others may occur during infancy, childhood, or adolescence [1,2].

The causes and types of birthmarks in children can be divided into the following key categories: inherited or acquired. The genetic basis means that children may inherit a tendency to develop certain types of moles [4].

Children exposed to intense sunlight are more susceptible to developing birthmarks. The sun can lead to an overproduction of melanin, which may manifest in pigment changes. Additionally, specific hormonal changes, such as those that occur during puberty, can influence the development of moles [4,11].

An anamnesis and physical examination of a child with skin lesions is essential in assessing their skin health. The anamnesis should include the following elements:

- Were the nevi present from birth, or did they appear later?
- Have the nevi increased in size or changed shape, color, or texture?
- Did any nevi bleed, itch, or hurt?
- Is there a family history of skin cancer?
- The examining doctor must remember the question about the child’s exposure to the sun.

The physical examination of the skin should begin with a general examination and assessment of the condition of the child’s skin. Then, each mole must be assessed separately according to the ABCDE rule. It is a good idea to take photos of the moles to document their appearance and track any changes in the future. The examination must extend the assessment beyond the areas where nevi are located. The child’s entire body should be assessed for other skin changes that may not be noticed by his or his parents. The skin on hairy areas such as the scalp, pubic skin, and mucous membrane must also be checked [7,8]. During palpation, the doctor should gently press fingers against the moles to assess their consistency and detect any invisible changes beneath the skin surface. It is essential to always pay attention to enlarged lymph nodes, which may suggest that the skin lesions are related to a more severe problem [7,8]. In case of any disturbing changes, it is worth consulting a dermatologist. Dermatoscopy allows us to precisely assess moles’ structure and pigmentation [7]. Taking photos of the skin lesion can help monitor the change over time and allow for a more detailed analysis [26].

If there is any doubt as to the nature of the lesion or if skin cancer is suspected, the doctor may order a biopsy. This procedure involves taking a small fragment of a skin lesion or performing an excisional biopsy that removes the entire lesion. The biopsy result ultimately allows us to accurately determine whether the lesion is benign or malignant [12,30,31]. Most changes are benign and do not require treatment. Situations in which nevus treatment is considered include cosmetic nevus removal, any disturbing changes, and in the case of large congenital nevi that have an increased risk of transformation. Moles are usually resected safely and minimally invasively, and a dermatology specialist or surgeon should decide to treat them after a thorough assessment. The treatment of melanomas in children is like that in adults. Still, there are some differences and exceptions due to the patient’s age and the children’s specific needs. In most cases, melanoma is treated by a surgical removal of the skin lesion, with margins depending on the stage of the lesion. A sentinel node biopsy may be necessary if the melanoma is more advanced or lymph node metastases are suspected [23,29]. In some cases, adjuvant therapy may be recommended in children with advanced melanoma after surgery. This may include immunotherapy or targeted treatment to reduce the risk of relapse. Radiation therapy may be considered for advanced melanoma or when surgery is impossible [32]. Chemotherapy is rarely used to treat melanoma because melanoma is poorly responsive to traditional chemotherapy methods. However, it can be used in some cases, especially in the advanced stages of the disease. Targeted therapies, which attack specific genetic changes in cancer cells, and immunotherapy, which enhances the immune system’s response to cancer, are becoming increasingly important options for treating pediatric melanoma [3]. Psychological support should be added to the treatment by a multidisciplinary oncologist, pediatric surgeon, and radiotherapist [23,29,32].

Prepubertal melanoma refers to melanoma that occurs in individuals who have not yet reached puberty, typically under the age of 12. Puberty changes the biology of melanocytes. Adult melanoma, on the other hand, refers to melanoma that occurs in individuals who have gone through puberty and are typically older, although there is no strict age cutoff [3,23,29]. Here are some key differences between prepubertal and adult melanoma. The first is incidence. Melanoma is rare in children, including those who have not yet reached puberty. The incidence of melanoma increases with age, with the majority of cases occurring in adults, especially those over the age of 50 [3,23,24,29].

The second difference is that risk factors for melanoma in children may differ from those in adults. While excessive sun exposure and a history of sunburns are significant risk factors for melanoma in adults, they may play a lesser role in prepubertal melanoma. In some cases, genetic factors or certain congenital conditions may increase the risk of melanoma in children.

Also, the subtypes of melanoma in children may differ from those seen in adults. For example, melanoma in children is more likely to be of the superficial spreading type, while nodular melanoma is more common in adults. The next feature is that the location of melanoma can be different in children and adults. In children, melanoma may occur in areas of the body that are less exposed to the sun, such as the trunk or extremities. In adults, melanoma is more likely to occur in sun-exposed areas like the back, chest, arms, and legs. There is also a difference in prognosis between prepubertal and adult melanoma.

Overall, melanoma in children tends to have a better prognosis than melanoma in adults. This may be partly due to differences in tumor biology and the aggressiveness of the disease. However, early detection and prompt treatment are essential for favorable outcomes in children and adults [3,23,24,32].

Healthcare providers must consider age-specific factors when diagnosing and treating melanoma in children versus adults, including differences in risk factors, clinical presentation, and treatment considerations. Regular skin examinations and sun protection practices are essential for all individuals, regardless of age, to help prevent melanoma and other skin cancers [3,23,24,29,32,33].

## 9. Take Home Message

Most nevi remain stable and do not undergo malignant transformation to melanoma. The risk of such a transformation within a year is estimated at less than 0.005% [4]. Although the risk of progression to melanoma from a single nevus is very low, it should also be remembered that in children, nevi, especially multiple, are a major risk factor for melanoma.

The early diagnosis of melanoma is critical, and it should be remembered that visual inspection or self-examination with the naked eye alone may not be enough. Melanoma may remain unnoticed, especially in regions such as feet, scalp, and genitalia, where even 2D or 3D whole-body sequential photography is unreliable. Dermoscopy also shows a different picture of nevi predisposing to melanoma, such as globular or reticular nevi.

Children, adolescents, and their caregivers should receive widespread counseling on prevention, such as sun-protective behavior and self-skin examinations. Information on the role of genetic and environmental factors in melanoma development and diagnostic methods, including sequential dermoscopic imaging and 2D or 3D whole-body sequential photography, should also be discussed in detail.

Regular skin examinations and sun protection practices are important for all individuals, regardless of age, to help prevent melanoma and other skin cancers.

## Figures and Tables

**Table 1:** Table summarizing similarities and differences in nevi in children.

Type of Nevus	Appearance	Color	Evolution	Risk of Malignancy	Common Sites	Histological Layers	Treatment
Congenital nevi	single, oval, presence of hair	brown or black, uniform in color	papillary or nodular surface	most congenital pigmented nevi do not become cancerous	trunk or limbs, although they can appear anywhere on the body	dermis and/or subcutis	observation; regular monitoring is recommended; skin protection
Acquired melanocytic nevi							
Benign pigmented nevus	regular, symmetrical structure, a well-defined border, and evenly distributed pigment	black, brown, skin-colored, pink, steel blue	flat or slightly raised	vast majority of moles do not transform into skin cancer	various parts of the body	different histological layers, primary epidermis and dermis	observation; regular monitoring is recommended; skin protection
Atypical (dysplastic) pigmented nevus	asymmetrical shape, size of more than 5 mm, irregular edge	variety of colors, brown, pink, and tan	flat, slightly raised, or bumpier surface	can develop into melanoma	various parts of the body, upper back, chest, scalp, butttocks, lower legs, palm and soles	different histological layers, primary epidermis and dermis	observation; frequent regular monitoring is recommended (every 6 months); excisional biopsy; skin protection
Nevus spilus	light brown macular background dotted with many darker spots or papules	light brown with darker spots	flat with bumpier parts	development of melanomas within diffuse nevi reported	various parts of the body, chest and upper extremities are the most common locations	different histological layers, primary epidermis, junctional zone and dermis	observation; regular monitoring; excisional biopsy; skin protection
Spitz nevus	papular pigmented nevus; usually single	brownish, red, bluish	papular pigmented	majority of Spitz nevi are benign and do not transform into melanoma, some atypical or unusual may exhibit features that resemble melanoma	various parts of the body, extremities, face, trunk, scalp, genital area	different histological layers, primary epidermis, junctional zone and dermis	observation; regular monitoring every 3–6 months/excision; skin protection
Sutton’s nevus	depigmented or hypopigmented ring or halo surrounding an existing nevus	central part typically pigmented and may vary in color, such as brown or black	smooth, slightly raised, or flat	generally benign	various parts of the body, trunk, arms and legs, shoulders	different histological layers, primary epidermis and dermis	observation; regular monitoring; skin protection
Vitiligo	patches or depigmented areas on the skin	patches or depigmented	flat	benign	various parts of the body	primary epidermis; dermis	observation
Becker’s nevus	large brown spot with increased hairiness, irregular borders	light brown to dark brown	smooth or slightly raised, hairy	does not pose a risk of transformation to melanoma	various parts of the body, upper body, shoulder, chest, upper arm, back	epidermis and dermis	observation; regular monitoring; skin protection
Blue nevus	maculopapular lesion the size of a lentil grain, borders typically well defined	blue	Smooth or nodular	“safe nevi”, which are rarely the starting point of melanoma	various parts of the body, scalp, face, hands, and feet	dermis, hypodermis	observation; regular monitoring; skin protection

## Data Availability

The original contributions presented in the study are included in the article, further inquiries can be directed to the corresponding author.
